# The development and application of performance indicators to assess veterinarians’ adherence to the clinical practice *Streptococcus suis* in weaned pigs guideline

**DOI:** 10.1186/s12917-025-04550-0

**Published:** 2025-02-25

**Authors:** Isaura Y.A. Wayop, Jaap A. Wagenaar, Emely de Vet, Anke Lambooij, David C. Speksnijder

**Affiliations:** 1 Division of Infectious Diseases and Immunology, Department Biomolecular Health Sciences, Faculty of Veterinary Medicine, Utrecht University, Yalelaan 1, Utrecht, CL 3584 The Netherlands; 2 Wageningen Bioveterinary Research, Houtribweg 39, Lelystad, RA 8221 The Netherlands; 3 Tilburg School of Humanities and Digital Sciences, University College Tilburg, PO Box 90153, Tilburg, LE 5000 The Netherlands; 4 The Dutch Institute for Rational Use of Medicine, Churchilllaan 11, Utrecht, GV 3527 The Netherlands; 5 University Farm Animal Clinic ULP, Reijerscopse Overgang 1, Harmelen, LZ 3481 the Netherlands

**Keywords:** Quality indicators, Key figures, Veterinary guidelines, Antimicrobial stewardship, Quality measures, Questionnaire, Guideline adherence, Swine veterinarians, RAND/UCLA method, Antimicrobial resistance

## Abstract

**Background:**

To combat antimicrobial resistance, initiatives have been launched worldwide to reduce antimicrobial use in humans and animals. In the Netherlands, the pig industry has made significant strides in reducing antimicrobial use, yet considerable variation exists in usage and prescription of antimicrobials between different swine farms and swine veterinarians. Clinical practice guidelines have been developed to support veterinarians to further reduce prescription of antimicrobials. In 2014, the *Streptococcus suis (S. suis)* clinical practice guideline was introduced. To date, no information has been collected about the extent to which veterinarians were using this guideline. Therefore, we developed performance indicators involving a six-step approach using a modified RAND/UCLA method aimed at assessing veterinarians’ adherence to the *S. suis* guideline. To support our results and to provide a more comprehensive understanding, we developed and circulated a questionnaire. The performance indicators and questionnaire were completed by 33 active swine veterinarians.

**Results:**

The final set of five performance indicators encompassed antimicrobial use, the ratio 1st to 2nd or 3rd choice antimicrobials, the argumentation for using 2nd choice antimicrobials, bacteriological examination including susceptibility testing, and the use of corticosteroids. In the questionnaire, 16 questions were included about veterinarians’ behavior linked to these five performance indicators. The results revealed a wide range in antimicrobial prescription among veterinarians dealing with *S. suis*-related issues on farms, suggesting that further improvement of antimicrobial stewardship is possible. Our findings show a discrepancy between the performance indicators based on observed data and veterinarians’ self-reported behaviors, particularly concerning the initiation of group treatments and the possibility that the advice provided by veterinarians may not always be consistently implemented in practice.

**Conclusions:**

The developed performance indicators on their own may not adequately reflect veterinarians’ adherence to the guideline, but collectively, they serve as a reliable indicator of adherence. By generating reliable and accurate outcomes, they complement self-reported behavior, which may be subject to unconscious self-report biases. Therefore, performance indicators are essential for use in intervention programs to measure veterinarians’ guideline adherence and should be incorporated into the development process of all clinical veterinary guidelines.

**Supplementary Information:**

The online version contains supplementary material available at 10.1186/s12917-025-04550-0.

## Background

Antimicrobial resistance is a global threat for human and animal health as it limits the effective therapeutic options to treat infections, thus becoming a leading cause of death. The widespread use of antimicrobials in animals is believed to be a substantial driver of antimicrobial resistance and, without appropriate actions, such resistance is expected to increase. Numerous initiatives have been launched globally, offering strategic approaches to address antimicrobial resistance [1–3].

In the Netherlands, antimicrobial use in animals decreased by 77.4% between 2009 and 2022 after the implementation of various regulations and measures [4, 5], followed by a reduction of resistance in indicator bacteria in food-producing animals [6]. Since 2012, the administration of all veterinary antimicrobials in the Netherlands is the responsibility of the veterinarians and antimicrobials are dispensed solely by veterinarians. Every professional farmer is required to have a contract with a veterinarian, establishing a one-to-one relationship. Pig farmers may stockpile a limited quantity of 1st choice (and sometimes 2nd choice) antimicrobials under strict conditions and initiate antimicrobial treatments to their animals which should be carefully recorded in their treatment records. The Netherlands Veterinary Medicines Institute (SDa) uses a benchmarking method for livestock farms that represents the acceptable use of antimicrobials and an action threshold for farms exceeding this level. The farm-level Defined Daily Dose Animal (DDDAF) is used to express the quantum of antimicrobials used at a farm. If farms exceed the action value, mandatory actions with the veterinarian or an advisory/quality team are necessary [7]. However, a wide variation in veterinarians’ antimicrobial prescriptions and in antimicrobial use at farms in all animal production sectors still exists. The policy of the Ministry of Agriculture, Nature, and Food Quality is to pay specific attention to farms with a (structurally) high use of antimicrobials so that they may be steered toward further reducing antimicrobial use [8].

The Netherlands has a very large pig industry with approximately 11.3 million pigs, and consequently a substantial volume of antimicrobials is used in this sector [9]. *Streptococcus suis (S. suis)* infections are seen as one of the major reasons for (high) antimicrobial use in the pig sector and specifically in weaned pigs [10]. The *S. suis* in weaned pigs clinical practice guideline (*S. suis* guideline) [11] was published by the Royal Dutch Veterinary Association in 2014 to assist (veterinary) practitioner decisions with the aim of improving antimicrobial stewardship (i.e., responsible use of antimicrobials). The guideline includes 49 pages and includes recommendations about the data-inspection, anamnesis, farm and animal group inspection, clinical examination of the piglets, bacterial examination, (antimicrobial) treatment plan, check and follow-ups, prevention, and prognostic expectations of *S. suis* infections in commercial pig operations. The *S. suis* guideline refers to the Dutch swine formulary for antimicrobial choices. Results of a survey conducted in 2016 [12] indicated that the *S. suis* guideline was used only partly or not at all by most practicing swine veterinarians surveyed, but the extent of use and the elements applied remain unknown. Despite the impressive decrease in antimicrobial sales in the Netherlands in 2019, 26% of the farms with weaned pigs have a usage above the 20 DDDA action threshold [13]. No materials have yet been developed to measure veterinarians’ adherence to, or improve the adoption of, the *S. suis* guideline.

Performance indicators (also referred to as quality indicators, quality measures, service indicators, or key figures) are measurable items referring to structures, processes, and outcomes of care [14]. Performance indicators are important tools for assessing the implementation of guideline recommendations and are often used in human medicine [15–18]. Evidence-based clinical practice guidelines are a frequent source for the development of performance indicators [14]. There is no golden standard for the development of guideline-based performance indicators, and the methodological approaches to guideline-based performance indicator development vary considerably [17–19]. In contrast to human medicine, not many countries have evidence-based veterinary clinical practice guidelines regarding antimicrobial stewardship, and existing guidelines are relatively new [20–22]. To our knowledge, no studies have investigated the development of performance indicators to assess adherence to such guidelines in veterinary medicine. Thirteen veterinary guidelines have been published in the Netherlands, but performance indicators have not yet been developed for any of them.

The aim of this study was to assess veterinarians’ adherence to the *S. suis* guideline through the development and utilization of performance indicators based on observed quantitative data. To support our findings and provide more context, we developed and utilized a questionnaire that provided data on veterinarians’ self-reported behavior regarding the *S. suis* guideline. This study is part of a larger project aimed at developing a theory-based intervention program to support swine veterinarians in adhering to the *S. suis* guideline and improving antimicrobial stewardship in practice [23].

## Methods

### The project team

The main activities for the development of performance indicators for the *S. suis* guideline were carried out by the project leader (IW, researcher and experienced clinical veterinarian) and a project member from the Dutch Institute for the Rational Use of Medicines (AL) with extensive experience in the development of performance indicators for pharmacotherapeutic audit meetings (peer meetings of general practitioners and pharmacists) regarding human medicine. The process was guided by the project team, which included specialists from various fields: veterinary practitioners, specialists in the development of performance indicators and peer-learning modules in human medicine, academic experts in veterinary clinical infectiology, qualitative research, general practice medicine, and health communication and behavior change.

### The performance indicators

#### Theoretical background

**Fig. 1:**
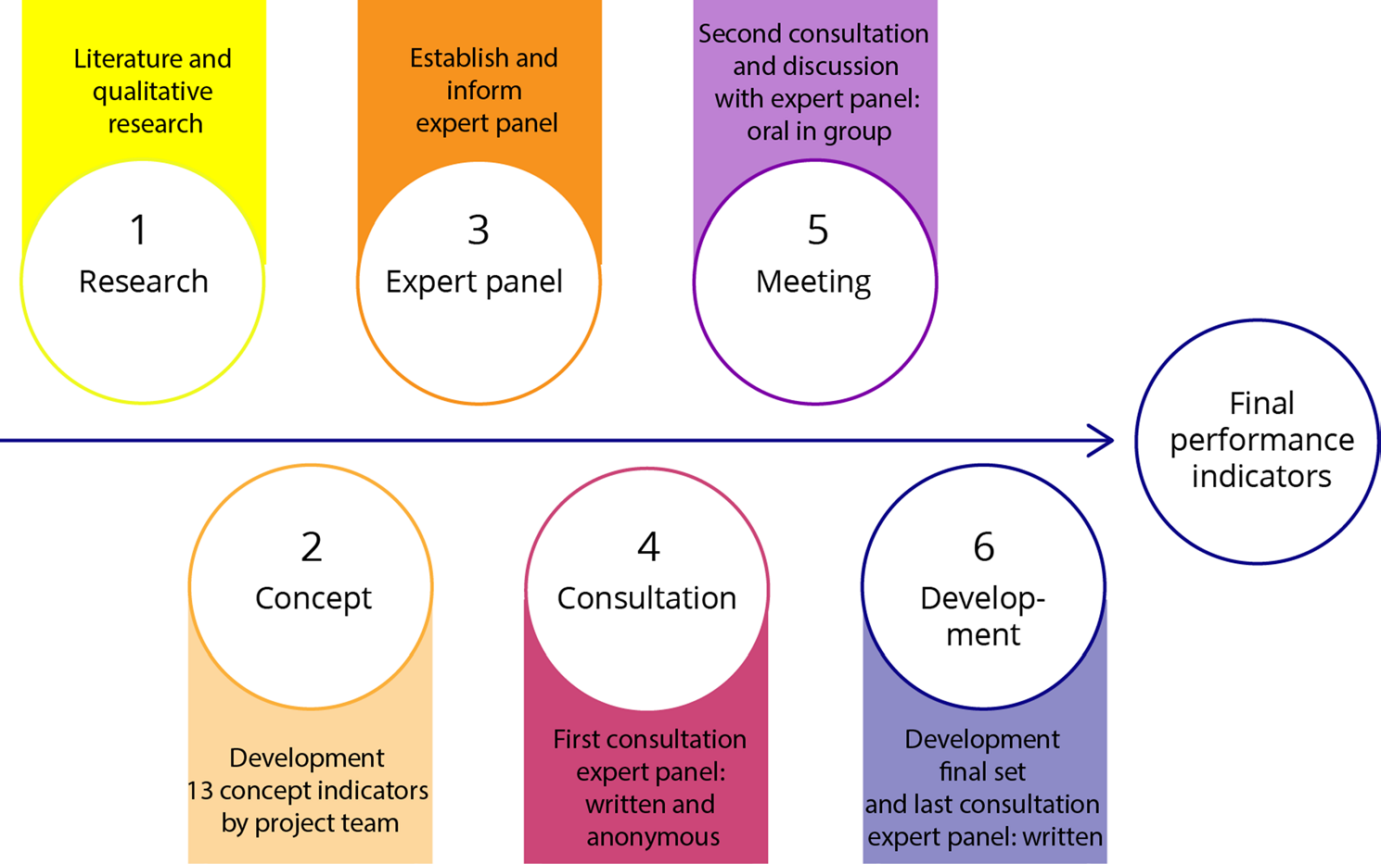
Development process performance indicators S. suis guideline

The developed performance indicators are inspired by the RAND/UCLA (modified Delphi) approach [24]. This method combines expert opinions and the best available scientific evidence to yield a statement regarding the appropriateness of performing a procedure and is used to develop validated, expert-endorsed performance indicators in a wide range of fields [17, 25]. We modified the steps of the RAND/UCLA method to the specific context of the *S. suis* guideline. A summary of the process is shown in Fig. 1.

#### Development process

The performance indicator development process took place between November 2018 and October 2019 and included six steps.

#### Step 1 literature and qualitative research

To determine all important parts and overall targets of the *S. suis* guideline, we engaged in the following three activities: (1) study and note important aspects of the *S. suis* guideline, (2) dialogue with the *S. suis* guideline developers and stakeholders (representatives of the two Dutch veterinary professional associations (focused on promoting of professional development and advocating for veterinarians), two European College of Porcine Health Management (ECPHM) diplomates (specialists), an SDa representative, a representative of the Foundation for Certified Veterinarians (SGD) (responsible for certifying farm animal veterinarians a license to practice) and the Dutch Animal Health Services (Royal GD)), and (3) interview the guideline adopters (13 practice swine veterinarians and five farmers) which is published separately [26]. No other guidelines or indicators around the clinical approach to *S. suis* infections in pigs were found during our literature search on PubMed and Google Scholar.

#### Step 2 project team develops conceptual performance indicators

Following the results of step 1, we listed ideas for performance indicators connected to important statements (recommendations) in the *S. suis* guideline. These ideas for performance indicators were further developed during three consultation rounds within the project team. Each consultation round started with individual written feedback on the performance indicators, followed by group discussion. Subsequently, a written report detailing the updated set of performance indicators was produced, which then served as the basis for the subsequent round of consultations. Three criteria were used to develop the performance indicators: (1) the importance of what is being measured, (2) the scientific soundness (validity) of the measure, and (3) the feasibility/costs of obtaining data to calculate the performance indicators [27]. In the last round, 13 conceptual performance indicators (see Supplemental Table 1) were identified, including discussion points and questions for the expert panel.

#### Step 3 establish expert panel

An independent expert panel was formed, comprised of four practicing swine veterinarians with varying levels of experience (between five and 30 years) and from different veterinary practices (each with between four and 14 certified swine veterinarians), along with one ECPHM diplomate. This panel was tasked with elaborating on the 13 conceptual performance indicators, following the model of expert panels in human medicine [28]. These performance indicators were presented to the panel.

#### Step 4 first consultation round expert panel: written and anonymous

The expert panel was asked to give written feedback on the three main questions: is this performance indicator feasible to measure in practice, how much time does it take to gather the information for this indicator, and which parts of the *S. suis* guideline are the most important to include in the performance indicators? Additional specific questions were asked about each indicator (see Supplemental Table 1). The expert panel was also asked if they had ideas for other performance indicators besides the existing 13, but they did not provide any new ideas or suggestions in addition to those. The project team prepared an anonymized feedback report and sent it back to the expert panel as preparation for step 5.

#### Step 5 second consultation round and discussion expert panel: physical group meeting

A physical meeting for the expert panel’s feedback on, and discussion of, the performance indicators was organized. Given the results from the first consultation round, the experts discussed the three criteria that the project team used in step 2. For all discussed performance indicators, experts expressed the importance of judging the outcome of a performance indicator in relation to other indicators and the farm’s context. A summary of the discussion points is given in Supplemental Table 1. More general overlapping points were also discussed (Supplemental Table 2) – for example, the quality of the *S. suis* guideline and other factors that the veterinarian could not influence directly, such as which recommendations are actually implemented by farmers.

No attempt was made to force the panel to consensus. Rather, the discussion established whether discrepant feedback was attributable to real clinical disagreements (“real” disagreement) or to misunderstandings (“artefactual” disagreements). Most frequently mentioned reasons for deeming a performance indicator inappropriate were that the indicator did not adequately reflect the veterinarian’s adherence to the *S. suis* guideline and concerns about the insufficient or inaccurate registration of information underlying the indicator. However, the panel did agree about the appropriateness of the final performance indicators.

#### Step 6 final consultation round expert panel: written

Five suitable performance indicators, including their limitations as discussed in the meeting, were concretely elaborated, including specific underlying calculations, and presented in a written report to the expert panel. The expert panel agreed upon these performance indicators and did not provide any additional feedback that would require modifications.

### Self-reported behaviors

A questionnaire was constructed as part of the development of an intervention program [23] and was piloted with a practicing swine veterinarian. The questions, which aimed to assess veterinarians’ self-reported adherence to the *S. suis* guideline, were informed by qualitative research [26]. The questionnaire included 63 items, of which 16 specifically addressed the veterinarian’s self-reported adherence to the guideline and related directly to the five final performance indicators (Supplemental Table 3). The remaining items related to other aspects (other performance objectives) of the intervention study and were not relevant for this study. The questionnaire development process took place between December 2019 and April 2020.

### Data collection and analysis

The project leader contacted veterinary practices with swine veterinarians in the Netherlands by email and phone to enquire about their willingness to participate in an accredited intervention program. A total of 56 swine veterinarians, reflecting the density of pig farms across the Netherlands, were invited to participate in the program. Part of the intervention program was the delivery of data to calculate the performance indicators and to fill out the questionnaire to assess self-reported behavior. The inclusion criteria for participation were being an active swine veterinarian and providing veterinary care to swine farms with *S. suis* problems in the Netherlands and Belgium. We used the definition of an *S. suis* problem farm as specified in the *S. suis* guideline: an *S. suis* problem farm is a farm where antimicrobial use to treat weaned pigs with clinical symptoms of *S. suis* results in a level of use above the threshold value (20 DDDA at that juncture) and/or the use of 2nd choice antimicrobials. The maximum of *S. suis* problem farms per veterinarian to be included in our study was five. If a participant had more than five *S. suis* problem farms under her/his care, (s)he could choose the five farms with the biggest *S. suis* problem in terms of their antimicrobial use for *S. suis* problems. In the Netherlands, veterinary antimicrobials are classified as 1st, 2nd, and 3rd choice antimicrobials, where 1st choice antimicrobials can be prescribed empirically, 2nd choice antimicrobials can be prescribed if it is well reasoned and documented (based on antimicrobial susceptibility testing, the history of antimicrobial resistance at the farm, or clinical need if a bacteriological examination is not possible), and 3rd choice antimicrobials can be prescribed to individual animals only after susceptibility testing because of antimicrobials’ importance for public health [29]. For *S. suis*, the swine formulary in the Netherlands recommends procaine benzylpenicillin and trimethoprim/sulfamethoxazole as 1st choice antimicrobials and amoxicillin and ampicillin as 2nd choice antimicrobials.

Of the 56 invited swine veterinarians, 33 filled in the questionnaire using an online survey platform (Qualtrics^XM^) and provided retrospective data from 125 *S. suis* problem farms to calculate performance indicators prior to the start of the intervention program. The survey was opened a week before the program and closed on the day the program started. From each farm, we collected: the total antimicrobial and other medicine usage (obtained from centrally registered prescription reports for all treatments in weaned pigs (not solely for S. suis treatments)), the argumentation for the use of 2nd choice antimicrobials, and the number and results of bacteriological examinations of postmortems (obtained from laboratory reports and veterinarians’ records). Supplemental Table 4 shows the number of participating farms per veterinarian, ranging from 1 to maximum 5 (for logistical reasons). The performance indicators data covered a 12-month period and were obtained with informed consent from the veterinarians for use in this research. Reasons for not providing data for the performance indicators were mostly lack of time to participate or that the swine veterinarian did not have *S. suis* problem farms. Of the 33 participants, four supervised their farms with the biggest *S. suis* problems in neighboring Belgium and could therefore not answer the questions or provide data about the use of corticosteroids, as there are different regulations about the use of corticosteroids in Belgium. One of the 33 veterinarians provided data for only three of the five performance indicators.

The performance indicators were calculated for each veterinarian individually, and descriptive statistics and correlations between the performance indicators and the answers to the questionnaire were computed. The correlation was performed using the Kendall method in R v 4.2.3 [30].

## Results

### Final performance indicators

Of the initial 13 conceptual performance indicators, the project team judged five performance indicators as appropriate, when applied as a set for *S. suis* problem farms, to measure veterinarians’ adherence to the *S. suis* guideline. These five performance indicators are: antimicrobial use, the ratio of 1st to 2nd or 3rd choice antimicrobials, the argumentation for 2nd choice antimicrobials, the bacteriological examination of *S. suis* derived from piglets at postmortems, and the use of corticosteroids in diseased piglets (Table 1). Examples of calculations of the five performance indicators can be found in Supplemental Table 5.

**Table 1:** Final performance indicators *S. suis* guideline

Performance indicator	Description of indicator	Relation to quality	Description formula
Antimicrobial use	Number indicating the relative deviation from the DDDA_F_ action value for weaned piglets in the Netherlands on selected *S. suis* problem farms over the previous 12 months. The DDDA_F_ represents the annualized count of days of antimicrobial drug use for a weaned pig (incorporating the standardized average weight of 17.5 kg in the DDDA_F_ formula) on the farm (7).	The main goal of the guideline was to increase antimicrobial stewardship principles, of which decreasing total antimicrobial use is part. The DDDA_F_ of weaned piglets on a farm indicates the average level of antimicrobial use per year. These antimicrobials were used for piglets with *S. suis* and piglets with other conditions (such as respiratory diseases and gastro-intestinal infections). Because we include only *S. suis* problem farms, we assume that most of the DDDA_F_ of weaned piglets on farms is caused by *S. suis* problems.	Nominator: Average DDDA_F_ of weaned piglets on the included *S. suis* problem farms (maximum five) as per the veterinarian. Denominator: Action value of the DDDA_F_ of weaned piglets in the Netherlands according to the most recent SDa report. Currently, the action value is set at 20 DDDA_F_. Outcome: Number from 0 to infinity (inclusive) representing the relative AMU compared to the action value. Under 1 means an average antimicrobial use under the action value, and above 1 means an average antimicrobial use above the action value, for weaned piglets.
The ratio of 1st to 2nd or 3rd choice antimicrobials	Percentage of weighted average of fractions of prescribed 1st choice antimicrobials relative to the DDDA_F_ of weaned piglets on selected *S. suis* problem farms, over the past 12 months.	The guideline advises maximizing the proportion of 1st choice antimicrobials used over 2nd and 3rd choice antimicrobials for weaned piglets with *S. suis.*	Nominator: The sum of DDDA_F_s times the 1st choice antimicrobials, so the relative use of 1st choice antimicrobials to 2nd and 3rd choice antimicrobials used for weaned pigs on the selected farms. Denominator: The sum of weaned piglets’ DDDA_F_s on the selected farm. The results are summed to calculate the outcome for the veterinarian and divided by 100. Outcome: Number from 0 to 1 (inclusive). Increase means relatively more use of 1st choice antimicrobials.
The argumentation for 2nd choice antimicrobials	The relative proportion of 2nd choice antimicrobial use supported by bacteriological examination, including an antibiogram and/or a report of the farm history by the veterinarian, to address the issue of the selected *S. suis* problem farms, over the past 12 months, with a maximum of four times per farm. In the report of the farm history, it must be stated that 1st choice treatment was applied but proved clinically ineffective.	The guideline recommends arguing the use of 2nd choice antimicrobials with an antibiogram or report as most *S. suis* isolates are susceptible to 1st choice antimicrobials in vitro. However, it also states that in vivo, efficacy may be insufficient. If the use of a 1st choice drug has proven ineffective in the recent past after therapy evaluation, and this is documented, then a 2nd choice antimicrobial can be chosen accordingly. Both the result of an antibiogram and a documented therapy evaluation are valid for three months according to the guideline. The data for calculation of this indicator are collected for the last four times a 2nd choice antimicrobial is prescribed, as the data collection is time-consuming.	Nominator: Number of times the prescription of 2nd choice antimicrobials for *S. suis* treatments was accompanied with results from an antibiogram or therapy evaluation arguing for this choice, over the past 12 months, for *S. suis* treatments, with a maximum of four times. Denominator: Number of times selected *S. suis* problem farms in the past 12 months used 2nd choice antimicrobials for *S. suis* treatments, with a maximum of four times per farm. Outcome: Number from 0 to 1 (inclusive). Increase means more adherence.
Bacteriological examination of piglets	The relative proportion of quarters in a year in which bacteriological examination (including susceptibility testing) was performed during post-mortems when examination was also indicated according to the guideline, over the past 12 months.	The guideline recommends post-mortem examinations on at least two clinical cases with characteristic *S. suis* disease symptoms twice a year, including bacteriological examination of infected organs and susceptibility testing, in the event of a first disease outbreak (the first occurrence of clinical *S. suis* infections after a period of no clinical cases), to confirm the *S. suis* diagnosis. For problem farms, the guideline recommends a frequency of bacteriological examination four times a year which equals once every quarter.	Nominator: The number of quarters in which post-mortem examinations including bacteriology on- at least two typical *S. suis* infected piglets occurred in the past 12 months. If more than two piglets were examined multiple times in one quarter, the number remains one (one count per quarter). If only one piglet has been examined in one quarter, this counts as a half. Denominator: The number of quarters in which pathological and bacteriological examination is indicated by the guideline in the past 12 months. This is indicated when oral antimicrobials are at least once used as a group treatment in the particular quarter for control of *S. suis*, or when the farm is characterized by the guideline as a *S. suis* problem farm. If the farm was a problem farm in the past 12 months, the denominator is four. Thus, the denominator can be up to four per farm. The quality indicator is calculated for each selected farm, then the average is calculated by summing the results and dividing them by the total number of selected farms. Outcome: Number from 0 to 1 (inclusive). Increase means more adherence.
Use of Corticosteroids	The relative proportion of corticosteroid use on the selected *S. suis* problem farms over the last 12 months.	The guideline recommends treating a piglet with early symptoms of *S. suis* infections with corticosteroids. In the available farm-level veterinary medicines use data, the use of corticosteroids is not linked to any specific condition. As a result, it cannot be established whether the corticosteroids prescribed are used for weaned piglets with *S. suis* or other diseases. Therefore, we linked the sales of corticosteroids to antimicrobial *S. suis* group treatments and assume that, if antimicrobial group treatments are necessary, there are also early *S. suis* infections in piglets that need to be treated with corticosteroids. Absence of corticosteroid deliverance on a farm around a time of group treatments indicates that piglets with *S. suis* presumably did not receive corticosteroid treatment.	Nominator: Number of quarters that both corticosteroids and antimicrobials for a S. suis group treatment were prescribed at least once for the weaned pigs at each selected farm. Corticosteroids prescribed within one quarter were counted as one treatment. The numerator cannot be greater than the denominator. Denominator: Number of quarters in which at least one group treatment has been applied for *S. suis* during the past 12 months. All treatments within one quarter count as one treatment (maximum one count per quarter). Thus, the denominator can be up to four per farm. Outcome: Number from 0 to 1 (inclusive). Increase means more adherence.

### Application performance indicators

Of the 33 participants, 12 (36%) identified as female and 21 (64%) as male. The median number of years since they graduated as veterinarians was 18 (ranging from 0 to 37), so they had diverse years of experiences. In terms of veterinary practice size, the median was 11 fulltime swine veterinarians (ranging from 1 to 19), and the median working hours per week as a swine veterinarian were 40 (ranging from 5 to 40), so the majority were employed fulltime.

**Fig. 2:**
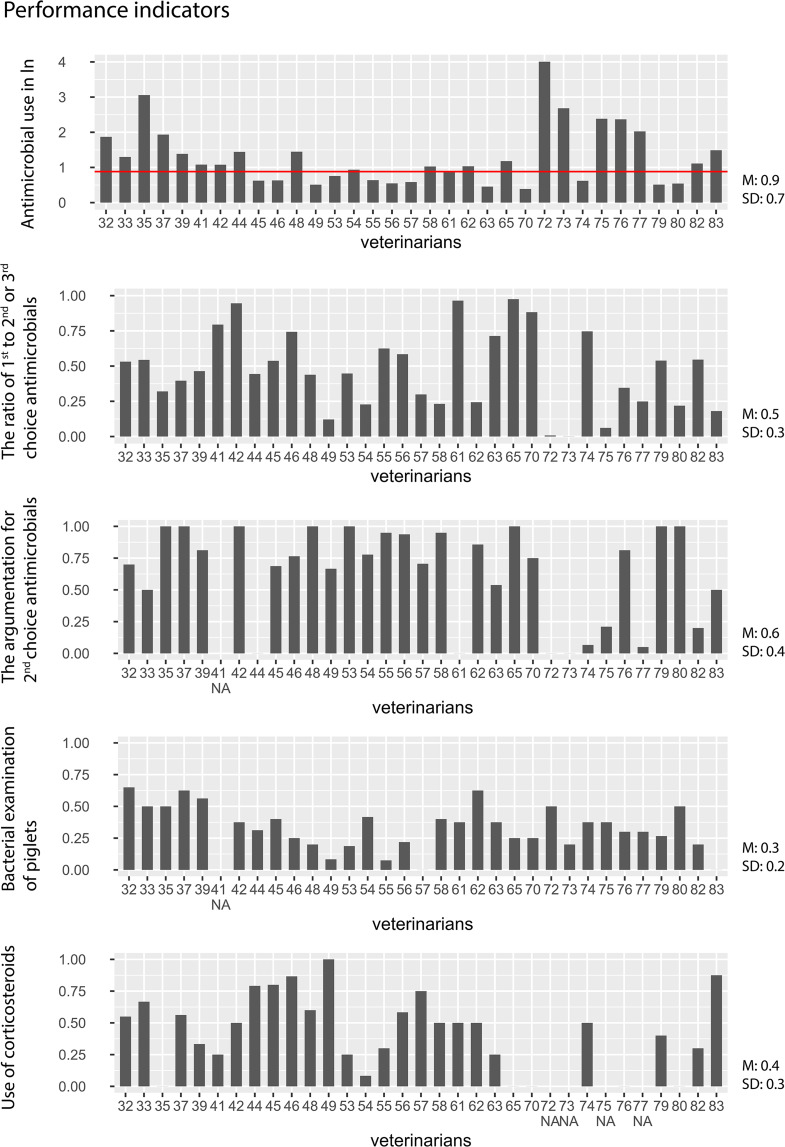
Results performance indicators. Thirty-three swine veterinarians provided data for five performance indicators. As there were some outliers, the results of the antimicrobial use performance indicator are showed in a natural logarithm (ln). The red line indicates the action value of the DDDA of weaned piglets in the Netherlands. A result of the performance indicator antimicrobial use below the red line suggests adherence to the S. suis guideline, whereas a result above the red line suggests less adherence to the S. suis guideline. The lower the figure of the performance indicators 1st choice antimicrobials, the argumentation for the use of 2nd choice antimicrobials, bacteriological examination, and corticosteroids, the lower the adherence to the S. suis guideline. The veterinarians’ numbers are their participant number in the study and therefore not sequentially ordered. Mean = M and Standard deviation = SD. Table 1 gives a full description of the five performance indicators

Of the participants, 52% (*n* = 17) had more than 25 swine farms under their care (ranging from 1 to 65), and 55% (*n* = 18) reported that they provided care to more than five *S. suis* problem farms (ranging from 1 to 26). Of the latter, only the five farms with the biggest *S. suis* problems per veterinarian were included in our study. The complete results of the participating veterinarians’ calculated performance indicators are shown in Fig. 2. Supplemental Table 6 shows the means and the standard deviation of the performance indicators.

The performance indicator, antimicrobial use, showed that 61% of the participants surpassed the recommended 20 DDDA_F_ action threshold on average and utilized larger quantities of antimicrobials on their selected *S. suis* problem farms. Additionally, 33.3% of them went beyond the action threshold by using at least twice the action value, averaging over 40 DDDA_F_, and there were also veterinarians with *S. suis* problem farms that used almost no antimicrobials on their selected problem farms.

The performance indicator ratio of 1st to 2nd or 3rd choice antimicrobials showed that almost half of the participants (45.5%) used more than 50% of the prescribed 1st choice antimicrobials. Two veterinarians did not use 1st choice antimicrobials on their *S. suis* problem farms, and three veterinarians used almost 100% 1st choice antimicrobials on their *S. suis* problem farms.

For the performance indicator, argumentation for 2nd choice antimicrobials, 25% of the participants scored the maximum score [1] and 53% had a score above 0.5, meaning that 78% of the participants more than half of the time correctly argued (supported by bacteriological examination, including an antibiogram and/or a report of the farm history which indicated 1st choice were not sufficient) for the prescription of the last four time they prescribed 2nd choice antimicrobials.

For the performance indicator, bacteriological examination, only 25% of the participants scored above 0.5, and none achieved the full score of 1, meaning none submitted the number of required animals for necropsy as the *S. suis* guideline indicated on all of their participating *S. suis* problem farms. More specifically, 75% of the participants submitted animals for necropsy less than half as frequently as recommended (four times a year for two clinical cases of *S. suis* in piglets).

For the final performance indicator regarding the use of corticosteroids, five participants scored the minimum score of 0, meaning that they did not prescribe them at all, and approximately half of the participants prescribed corticosteroids at, at least, half of the recommended frequency (score > 0.5) when prescribing antimicrobial group treatments for *S. suis.* One participant scored the maximum score of 1, demonstrating that it is possible to attain the full score for this indicator.

### Self-reported behaviors

**Fig. 3:**
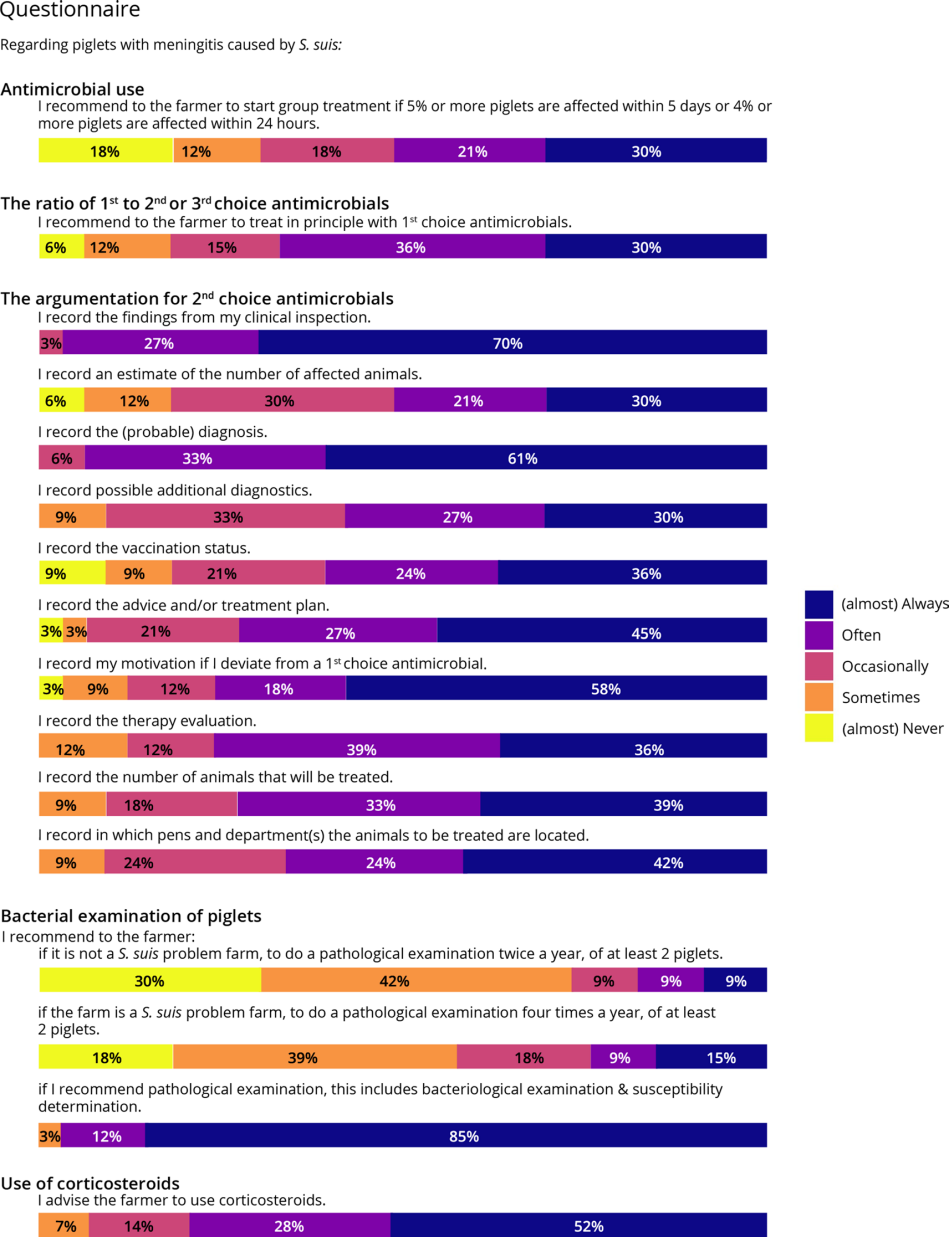
Results 16 questions related to performance indicators

The responses of 33 veterinarians, who also provided the data for these performance indicators, were analyzed. The complete results of the 16 questions linked to the five performance indicators are shown in Fig. 3. The means and the standard deviation of the individual questionnaire items are presented in Supplemental Table 6.

The guideline states that group treatments for *S. suis* need to be started when 5% or more piglets become infected within five days or when 4% of piglets become infected within 24 h. More than 50% of the participants reported doing so, but 18% reported doing this (almost) never, indicating that they start group treatment at another juncture. This could indirectly cause a higher antimicrobial use. Almost 70% of the participants reported starting treatment of piglets with *S. suis* in principle with 1st choice antimicrobials, and only 18% do this sometimes or (almost) never. A large fraction of the participants reported recording almost all information (findings clinical inspection, number affected animals, (probable) diagnosis, additional diagnostics, vaccination status, advice and/or treatment plan/ motivation for deviation 1st choice, therapy evaluation, number of animals treated, and pens and departments of treated animals) regarding *S. suis* problems at farms as stated in the *S. suis* guideline. Almost all participants (97%) reported that, if they recommended a pathological examination, bacteriological examination and susceptibility determination was performed. There was a wide variety in the frequency of bacteriological examination reported, as 24% do this often or (almost) always four times a year for at least two piglets for an *S. suis* problem farm and 57% do this (almost) never or sometimes. Nearly all participants (94%) reported occasionally, often, or (almost) always advising farmers to use corticosteroids for piglets with meningitis caused by *S. suis*.

### Correlation performance indicators – questionnaire

Of the 16 questions, three had a positive correlation with their corresponding performance indicator. The two performance indicators, antimicrobial use and the use of 1st choice antimicrobials, were not correlated with veterinarians’ self-reported estimations of when to initiate group treatments and to start primarily with 1st choice antimicrobials. The results show a moderate correlation between the argumentation for 2nd choice antimicrobials performance indicator and the question about the recording of the (probable) diagnosis (*r* = 0.3, *p* = 0.17) and therapy evaluation (*r* = 0.4, *p* = 0.15), and between the bacteriological examination of piglets performance indicator and the self-reported answers about the frequency of pathology examination (*r* = 0.3, *p* = 0.06). We found a significant correlation between the use of corticosteroids performance indicator and the self-reported answers to advise corticosteroids (*r* = 0.5, *p* = 0.01). The complete correlation results are given in Supplemental Table 6.

## Discussion

Using a modified RAND/UCLA (Delphi) approach, we systematically developed a set of five performance indicators for the guideline *S. suis* that demonstrated their applicability and value in clinical veterinary practice. The results indicate that there is still considerable room for improvement in adherence to the *S. suis* guideline, although the outcomes for the individual veterinarians are very diverse. The results point to some important leverage points for intervention programs addressing adherence to veterinary guidelines aiming to improve quality of veterinary care and antimicrobial stewardship.

Performance indicators are already widely used in human medicine, for example in audit and feedback meetings, for benchmarking, and also for accreditation of general practitioners [31, 32]. The literature describes improvements to quality of care attributable to the use of performance indicators [17]. The present study provides first indications that veterinary medicine also might benefit from the use of performance indicators. The following considerations need to be discussed regarding our five final performance indicators.

### Antimicrobial use

In this performance indicator, antimicrobial use for all infections in weaned pigs was assessed, not exclusively focusing on *S. suis* infections. Veterinary antimicrobial use in the Netherlands is not centrally registered in relation to the specific clinical indication for which it is prescribed. Because our selection comprised only *S. suis* problem farms, selected through their veterinarians after the definition of an *S. suis* problem was given, it was assumed that most antimicrobial use in the weaned piglets in the included farms was attributed to *S. suis* issues. We feel confident that this was a correct decision as *S. suis* is a common disease in the pig industry [33]. Swine veterinarians are familiar with the disease, and from qualitative research it is known that veterinarians know which farmers are using antimicrobials for *S. suis* infections [26]. However, it is crucial to note that, if there is an outbreak of another disease, antimicrobial use may be directed toward that specific infection. This highlights the ongoing importance of considering the right context in order to avoid misinterpretations and/or improve the administration with reasons for antimicrobial prescription.

### The ratio of 1st to 2nd or 3rd choice antimicrobials

This performance indicator utilizes a weighted average to incorporate the total antimicrobial volume, ensuring its impact on the overall result. Nevertheless, it remains plausible that a reduction or an increase in antimicrobial use within an antimicrobial category can exert a significant influence on the outcome. If a farm has an overall low antimicrobial use (below approximately 5 DDDA_F_), the introduction of just one prescription of a 2nd choice antimicrobial can yield substantial differences in the outcome of this performance indicator. Because we included only *S. suis* problem farms, we assumed that such changes would be minimal. This also underscores the importance of developing a set of indicators, rather than relying on single indicators, to better capture and understand the outcomes, as also described in the literature [34].

### The argumentation for 2nd choice antimicrobials

In this performance indicator, we focused on the last four episodes for which a farm used a 2nd choice antimicrobial, primarily because of the time-intensive nature of data retrieval. Therefore, the outcomes of this indicator can represent varying percentages of each veterinarian’s argumentations. To mitigate this variability, a more comprehensive understanding could be achieved by measuring this indicator multiple times over an extended period, contextualizing the results with those of other indicators, as is also previously mentioned, or broadening the scope of data collection to make more measurements easily possible.

### Bacteriological examination of piglets

Although we gave a complete description of the requested data including examples, overcomplete data were supplied. Some participants counted other methods such as laboratory diagnostics on saliva or blood for other (co-)infections, mostly because not all veterinarians interpreted certain terms (additional diagnostics and bacteriological examination) as referring to “only *S. suis”*. The importance of consensus on definitions to data quality is also described in human medicine [35]. Our lack of consensus about definitions was fortunately identified and discussed, but it was necessary to pay close attention to ensure that correct data were provided. For future data collection for performance indicators, the definition list could be expanded and discussed in the introduction meeting to prevent this as much as possible.

### The use of corticosteroids

In this performance indicator, we focused on corticosteroid prescriptions specifically for weaned pigs, without accounting for prescribed volume or considering other animal groups. It is acknowledged that corticosteroids may also be prescribed for other animal categories, such as sows, and subsequently utilized in weaned pigs, potentially leading to an inaccurate estimation in this performance indicator. However, we assumed that veterinarians are aware of such instances and can provide accurate data, as also happened in a few occurrences in our dataset. It is important to note that, depending on the magnitude of an *S. suis* outbreak, a single sale of corticosteroids could theoretically cover multiple group treatments. Nonetheless, our data did not reveal a significant volume of corticosteroid sales indicating such extensive usage.

Overall, it is important to note that our study investigated exclusively a selection of farms with the biggest *S. suis* problems and subsequent high antimicrobial use according to veterinarians. For some veterinarians, the indicators represent data originating from only one *S. suis* problem farm, whereas for others the indicators represent averages across a maximum of five farms. It is worth noting that the participating veterinarians managed up to 26 *S. suis* problem farms. The reliability of the indicators is enhanced when considered in the broader context of multiple farms, providing a more robust average. Nevertheless, prior to initiating this study, we were unaware of the anticipated number of *S. suis* problem farms for each veterinarian as no official records on this exist. Alongside the discovery that some veterinarians were associated with more than five *S. suis* problem farms, we observed that many veterinarians had no *S. suis* problem farms. These veterinarians were subsequently excluded from this study. Nevertheless, these veterinarians (can) still participate in intervention programs supporting veterinarians’ adherence to the *S. suis* guideline.

As discussed earlier, conceptual performance indicators were deemed inappropriate or adjusted because data collection was either impossible (unreliable) or very time-consuming. However, overall, data collection for all five performance indicators was still time-consuming, varying depending on the farm (size and number of problems) and the veterinarian (data collection skills). In this dedicated study, a lot of help was available and also financial compensation for time invested in data retrieval. In practice, it would be very helpful if the data were easier to retrieve and reuse, as is also suggested in previous studies in veterinary medicine [36] – for example, by including specific software packages in veterinarians’ Practice Management Systems so that information can be easily collected and anonymized or to ensure that veterinary assistants can collect the data. In human medicine, practitioners link codes (International Classification of Primary Care codes) for indications (symptoms and diagnoses) to codes for medicines (Anatomical Therapeutic Chemical codes), and this information is used for better patient care, education, and research [37–39]. Although veterinary medicines in the Netherlands also have codes, they are not administratively linked to specific animal diseases, which could be a helpful innovation in veterinary medicine too to serve the same purposes. Such developments would call for periodic re-evaluations of all existing and rejected (because of poor measurability) performance indicators. Performance indicators bear the risk of having a range of unintended and dysfunctional consequences that we need to prevent, such as misinterpretation, creation of perverse incentives, and increased administrative tasks [14, 40].

As this is the first time that veterinarians’ adherence to the *S. suis* guideline has been identified, we do not have other literature to confirm our results, other than the results of the 2016 survey [12]. Our performance indicators are not yet sufficiently validated to use in a system in which veterinarians are rewarded based on their results (pay-for-performance program). It is known that the drivers of swine veterinarians’ antimicrobial use are complex and diverse [26, 41]; this corresponds with our results. We also know that veterinarians can learn from one another via diverse approaches without negative consequences for animal welfare, health, and/or economic results [26, 42, 43]. In our study, a huge difference in the number of *S. suis* problem farms supervised by the different participating veterinarians was noticed. This observation may suggest that certain veterinarians either attract more problem farms or encounter difficulties in resolving *S. suis* issues, whereas others do not. This could be a consequence of the business model of the veterinary practice in which the veterinarians are working but could also have other explanations (such as their geographical area or farm sizes); this requires further research. The wide variety in antimicrobial use and the ratio of 1st and 2nd or 3rd choice antimicrobials underscores the potential for antimicrobial reduction and a shift to more use of 1st choice antimicrobials. Intervention programs, including peer learning [44, 45], could be helpful to show this variety to swine veterinarians and aid in reducing antimicrobial use at *S. suis* problem farms. During intervention programs, veterinarians can contextualize the outcomes of the performance indicators to prevent misinterpretations, as discussed earlier. This approach ensures that the outcomes of the performance indicators are not used simply as grading criteria but are instead utilized for comparing, evaluating, and discussing veterinarians’ work, as in peer-learning activities, mirroring practices in human medicine [37, 46].

The results of the argumentation for 2nd choice antimicrobials indicator show that a 100% score on this indicator is possible and that the majority of veterinarians already argue well (through bacterial examination or reports, see Table 1) for the use of 2nd choice antimicrobials. However, some swine veterinarians could make improvements on this indicator, and an up-to-date administration with complete visit reports could be helpful in achieving this. Because a correlation was found between this performance indicator and the self-reported behavior of reporting the (probable) diagnosis and therapy evaluation, we can suggest that these two aspects are important for a good argumentation. However, it is known that experienced veterinarians do not always prioritize administrative tasks [26] and that veterinarians with a relatively high antimicrobial prescription level have more difficulties with forming a realistic view of their antimicrobial use [43]. Highlighting the significance of comprehensive reports and enhancing administrative skills could enhance the outcomes of this indicator and foster a more accurate representation of veterinarians’ antimicrobial usage.

The results of the bacteriological examination of piglets indicator show that the statements in the *S. suis* guideline about bacteriological examination are poorly adopted or impossible in practice. The wide variety in the veterinarians’ self-reported behavior about the frequency of bacteriological examination suggests that there are many different or no protocols and approaches at veterinarian and veterinary practice level, as also found in other studies [26]. Following our results and previous studies, we can also suggest that some swine veterinarians find the frequency of bacteriological examination as stated in the *S. suis* guideline too high to achieve in practice and sometimes even impossible because of the absence of clinically diseased piglets when veterinarians are visiting the farms [12, 26]. There is a possibility that the statements in the *S. suis* guideline may need adjustment. Alternatively, the definitions in the guideline might require greater specificity, particularly regarding frequency, to align with the presence of clinically diseased piglets. However, highlighting to the veterinarians the significance of bacteriological examination for *S. suis* could enhance the outcomes of this indicator.

The results of the corticosteroids indicator show that the veterinarians’ adherence could be improved, and the results of the questionnaire show that the majority reported advising the use of corticosteroids. The corticosteroids performance indicator and the veterinarians’ self-reported behavior were correlated, suggesting that, if the swine veterinarians advise corticosteroids, this is adopted by farmers in practice. However, the reported high level in the questionnaire compared with the average level of the performance indicator suggest a self-report bias. Self-report bias is the deviation between the self-reported and the true values of the same measure [47]. Such a self-report bias might result from difficulties in estimating own performance, unawareness of own performance or an optimistic bias (i.e., the notion that generally individuals feel that they perform better than others).

Difficulties in estimating own performance could also be a reason why we did not find a correlation between other indicators (antimicrobial use and ratio 1st, 2nd, 3rd choice antimicrobials) and related questions. Other possibilities include that: (1) the veterinarians did not know exactly how they really performed in practice, as is also known from other literature (a proactive approach is necessary to login to a digital portal and see their antimicrobial use) [43, 48] and found in our qualitative research study [26]; (2) the veterinarians’ advice was not (always) adopted in practice by farmers; and (3) that contextual factors at farm level overruled veterinarians intentions when to start group treatments. The use of performance indicators demands greater resources and time compared with questionnaires, but they effectively mitigate self-report biases commonly associated with questionnaire-based assessments, ensuring more precise and reliable data as they are based on objective/observed data instead of self-reported data. However, the questionnaire did provide evidence on practice, attitudes, and knowledge, and combining them with the quantitative results of the indicators provided a greater insight into the realities of clinical veterinary practice, as is also known from the literature [49].

The development of our performance indicators could contribute to the creation of more performance indicators for other guidelines. Although existing Dutch veterinary clinical practice guidelines vary significantly and are applicable to diverse animal species across different sectors, our approach can be readily replicated and adjusted as needed. The fundamental steps remain consistent, and, given the insights gained from each specific guideline, we are now better equipped to anticipate challenges in data collection. For new veterinary clinical practice guidelines (nationally and internationally), our approach could be adapted and implemented to seamlessly integrate the guideline with corresponding performance indicators. This is a common approach in human medicine and ensures a better integration [17–19]. If the *S. suis* guideline is updated or if other significant developments occur (e.g.: a new vaccine or new regulations), it will be necessary to evaluate the current five performance indicators to determine whether adjustments are necessary [50–52].

## Conclusion

For any performance indicator, there are pros and cons, but, when used as a set, they appear to assess adequately veterinarians’ adherence to guidelines. Placing them in the right context is essential, as they are important tools for intervention programs. Although using a (modified) RAND/UCLA approach to develop performance indicators and incorporating them into peer-learning activities are common in human medicine, they are relatively new in veterinary medicine. To gain greater insights into veterinarians’ behavior, it is advisable to combine the results of the performance indicators with self-reported behavior. This approach prevents unconscious self-report bias, provides evidence for the adoption rate of veterinarians’ advice in practice where relevant, generates reliable and accurate outcomes, and adoption rates of guidelines. Our performance indicators for the *S. suis* guideline revealed a wide variety among veterinarians in their approach to *S. suis* problems on farms, and this could be utilized in an intervention program. Enhancing the performance indicators through this intervention program could optimize antimicrobial stewardship principles among swine veterinarians.

## Electronic supplementary material

Below is the link to the electronic supplementary material.

Supplementary Material 1

Supplementary Material 2

Supplementary Material 3

Supplementary Material 4

Supplementary Material 5

Supplementary Material 6

## Data Availability

The datasets used and/or analyzed during the current study are available from the corresponding author on reasonable request.
