# Supplementary material for: The development and application of performance indicators to assess veterinarians’ adherence to the clinical practice Streptococcus suis in weaned pigs guideline
Source: BMC Vet Res. 2025 Feb 25;21:101. doi: 10.1186/s12917-025-04550-0 (PMC11854134; doi:10.1186/s12917-025-04550-0)
Supplement: Supplementary file 1 — Supplementary Material 1 [file 12917_2025_4550_MOESM1_ESM.pdf]

Supplementary Table 1 Conceptual performance indicators *S. suis* guideline

PERFORMANCE INDICATOR	BACKGROUND AND RELATION TO QUALITY	DEFINITION INDICATOR AND QUESTIONS TO EXPERT PANEL (BESIDES MAIN QUESTIONS)	SUMMARY DISCUSSION POINTS AND FINAL DECISION
**1. Knowledge of *S. suis* guideline**	In principle, we assume that all swine veterinarians are aware of the existence of the *S. suis* guideline for weaned piglets. The question is to what extent the veterinarians are familiar with the content and specific recommendations of the guideline. It is worth noting that a veterinarian can act upon a *S. suis* outbreak according to the guideline even without being familiar with the guideline itself.<br><br>Complete knowledge of the guideline is necessary for full compliance with the guideline in practice. If the veterinarian is familiar with the following recommendations, it is assumed that (s)he has sufficient knowledge regarding all the recommendations in the guideline:<br>• Criteria to start group treatment of 5% diseased animals within five days or 4% diseased animals within 24 hours;<br>• Immediate actions to be taken: providing water + internal biosecurity (+ isolation of diseased pig);<br>• Pathological examination + bacteriological examination: two typical representatives twice a year + in case of problem farms, four typical representatives four times a year.	Proportion of the extent of the veterinarian's knowledge on the following three recommendations in the guideline: 1. when to advise group treatments, 2. the direct measures that need to be taken, and 3. when and how many times bacteriological examination is required.<br><br>*Can we test with this indicator whether a veterinarian is familiar with the* S. suis *guideline?*<br><br>*On what recommendations should we focus to get a good understanding?*	Value: this process indicator measures adherence to guideline for only three points. The extent of these three points is debated. It is mentioned that a veterinarian needs to know at least 10 risk factors (of the 15 mentioned in the guideline) but that these risk factors are seen as common knowledge for experienced swine veterinarians.<br><br>Registration and measurability: indicator is measurable by a questionnaire.<br><br>Others:<br><br>The advice about when to advise group treatments instead of individual treatments is questioned. This does not influence the measurement of the indicator for the veterinarian's knowledge, but it does for attitude. Because attitude plays a big role, the registration and outcome from this indicator can be false.<br><br>Indicator is considered inappropriate.

**2. Problem definition meningitis**	The guideline for *S. suis* is established for *S. suis* types only where piglets with neurological symptoms are observed. Step 1 in the guideline's flowchart is the problem definition "neurological symptoms in weaned piglets". If the answer is no, a new problem definition needs to be established. If the answer is yes, the veterinarian proceeds further in the flowchart. In practice, it is possible to choose to proceed further in the flowchart even though no neurological symptoms are observed in the weaned piglets.<br><br>The presence of weaned piglets with brain symptoms indicates whether a correct problem definition has been established. This is directly related to proper use of the guideline.	Percentage of piglets diagnosed with brain symptoms where action was taken according to the recommendations in the *S. suis* guideline in the past six months per farm.<br><br>*Did the veterinarian record whether animals with neurological symptoms were present?*<br><br>*Is this indicator practically feasible?*	Value: this process indicator measures adherence to, and implementation of, the guideline incorrectly.<br><br>Registration and measurability: indicator is not measurable. No reliable registration. Too much dependence on farmer registration at the farm.<br><br>Others:<br><br>It is questioned whether using the guideline for arthritis is always negative and how important the outcome from this indicator is in practice.<br><br>If possible, this indicator will measure knowledge and not attitude, which plays a role in adherence and can influence the outcome.<br><br>Indicator is considered inappropriate.
**3. Bacteriological examination**	The guideline advises conducting pathological examinations four times a year on a problem farm, with a minimum of two typical representatives each time. On a farm experiencing an initial disease outbreak, the recommendation is to conduct pathological examinations twice a year on two typical representatives.<br><br>Indicator is further developed, see results section of article.	The relative proportion of the frequency and numbers of typical representatives of *S. suis* infection for pathology, including bacteriological examination and susceptibility testing, during the past 12 months in accordance with the indications in the guideline.<br><br>*Is 12 months the right amount of time for this indicator?*<br><br>*Do we need to separate the frequency in a year from the number piglets in a year?*	Value: this outcome indicator measures adherence to the guideline for bacteriological examination correctly.<br><br>Registration and measurability: the indicator is properly measurable. Reports from examinations can be used. Reports from official laboratories but also from own laboratories can be used and are administrated.<br><br>Others:<br>This indicator does not include other diagnostic options, although this can also be good veterinary practice.<br><br>The frequency of bacteriological examination advised in the guideline is questioned. This does not influence the measurement and outcome of the indicator.<br><br>The difference between a first outbreak and an *S. suis* problem farm in practice is questioned. It is mentioned that almost all *S. suis* problem farms are familiar with *S. suis.*<br><br>Indicator is considered appropriate.

**4. The foundation for the use of 2nd choice antimicrobials**	In note 18 of the guideline, it is stated that there are differences between bacteriological susceptibility on the plate (in vitro) and clinical effectiveness (in vivo), emphasizing the importance of evaluating treatment outcomes. For this reason, no choice has been made of an indicator for treatment based solely on bacteriological examination and sensitivity testing. However, the farm history must be documented if another treatment is chosen.<br><br>Indicator is further developed, see results section of article.	The relative proportion of the use of 2nd choice antimicrobials is reasoned based on bacteriological examination including an antibiogram and/or a report of the farm history for the selected *S. suis* problem farms in the past 12 months.<br><br>*What is the incidence of the use of 2nd choice antimicrobials? Is 12 months a good time period?*<br><br>*How much time does it take to collect these data?*	Value: this outcome indicator measures therapy evaluation and bacteriological examination correctly.<br><br>Registration and measurability: indicator is measurable through collection of laboratory reports. This is time intensive. Therefore, the amount of time needed to measure the data must be proportional to the amount of time that is available in practice.<br><br>Indicator is considered appropriate.
**5. Direct measures**	As the first step in the treatment plan outlined in the guideline, the immediate measures to be taken are listed. These include: administering water (twice a day); internal biosecurity; isolation of the sick animals.<br><br>If the guideline recommendations are not followed, the guideline is not fully followed. Following step 1 of the treatment plan needs to be standard for all farms and is directly related to following the guideline.	Percentage of livestock farmers implementing the immediate actions to be taken in the event of an outbreak of S. suis.<br><br>*Is the outcome of this indicator influenced by the veterinarian or the farmer? Is it wise to judge only whether the veterinarian gave the right advice?*<br><br>*Is this practically measurable?*	Value: this outcome indicator does not measure the veterinarian's adherence to the guideline part of direct measures correctly.<br><br>Registration and measurability: indicator is not measurable. There is no reliable registration of this kind of measure.<br><br>Others:<br><br>In practice not feasible. The farmer needs to do this. In theory, it is possible to measure the veterinarian's advice, but this will be only administrative.<br><br>Indicator is considered inappropriate.

**6. Preventive measures**	Regardless of how the flowchart is followed, preventive measures must always be taken. A significant portion of the guideline focuses on the importance of thorough history-taking and diagnostics to arrive at the appropriate preventive measures for each farm. Additionally, a checklist of management measures is included in the S. suis guideline for this purpose.<br><br>If preventive measures are not taken, the *S. suis* guideline is not followed. This advice is strongly emphasized in the guideline; this points out the importance of this indicator.	Percentage of livestock farmers who implemented preventive measures after an outbreak.<br><br>*The preventive measures in the guideline are quite general. Which one do we consider the most important?*<br><br>*Can we align with the checklist from the guideline?*<br><br>*Is using this checklist important for the quality of the veterinarian?*<br><br>*Could veterinarians who do not use the checklist forget components more often, possibly due to familiarity with the farm? If so, then the indicator could be adjusted based on the percentage of checklist usage*.	Value: this outcome indicator can correctly measure whether preventive measures are being conducted. It is questioned what the number of preventive measures need to be (linked to an *S. suis* problem), but therefore the indicator can be adjusted to an infinite number instead of a percentage.<br><br>Registration and measurability: this indicator is measurable but the veterinarian's registration/reports need to be complete, and data collection is time intensive. The reports need to be analyzed and the data need to be reported to measure the indicator. This demands a lot of time and effort on the part of the veterinarian.<br><br>Others:<br><br>Preventive measures are seen as important for adherence to the guideline.<br><br>Veterinary antimicrobial use is not centrally registered in relation to the specific clinical indication for which it is prescribed. The preventive measures in the guideline are common/general measures. This questions the value of this indicator.<br><br>Indicator is considered inappropriate.

**7. Autovaccination**	The *S. suis* guideline discusses the use of autogenous vaccines but also mentions that the guideline working group is cautious regarding (autogenous) vaccination. However, a clear recommendation is provided that, when autogenous vaccines are used, the isolated *S. suis* strains should be serotyped four times a year on problem farms.<br><br>The guideline recommends that, when autovaccines are used, the isolated *S. suis* strains should be serotyped. In a problem farm, it is recommended that this be done four times a year. If this is not done, then the guideline is not followed.	The number of times strains of *S. suis* are serotyped if vaccination is carried out on the farm.<br><br>*How often are autovaccines used in practice and is there a standard approach?*	Value: this outcome indicator measures adherence to the S. suis guideline inaccurately. Although the guideline includes this statement, the guideline advises reserved use of autovaccines.<br><br>Registration and measurability: this indicator is measurable as the serotyping is reported.<br><br>Others:<br><br>Some farmers vaccinate sows and other the piglets, and this needs to be included in the indicator.<br><br>If the autogenous vaccine is successful, there is no reason/motivation to do bacteriological examination on piglets<br><br>Indicator is considered inappropriate.
**8. First choice antimicrobials**	The guideline states that the use of a 1st choice antibiotic should be the goal. If there is poor treatment success based on bacteriological examination or the farm history (i.e., treatment failures), deviation from this 1st choice antibiotic is possible if reported to the relevant authorities.<br><br>Indicator is further developed, see results section of article.	Percentage of weighted average of fractions of prescribed 1st antimicrobials relative to the DDDA of weaned piglets in the last 12 months on selected *S. suis* problem farms.<br><br>*How many months are sufficient?*	Value: this outcome indicator measures the veterinarian's adherence to the guideline but also includes other diseases.<br><br>Registration and measurability: this indicator is measurable, but there is no registration for *S. suis* separately.<br><br>Others:<br><br>Needs to be linked to total antimicrobial use to prevent false positive outcomes (e.g., high percentage 1st choice but also high total use of antimicrobials).<br><br>Indicator is considered appropriate.

**9. Argumentation for group treatment**	The guideline states that deciding when to switch to antimicrobial group treatments instead of individual therapy is not straightforward. The general advice is to be cautious about prescribing group treatments. The emphasis should be on prevention. The following criteria are set out in the guideline for initiating antimicrobial group treatments:<br>• 5% of weaned piglets in a herd/pen have become sick within five days;<br>• 4% of weaned piglets in a herd/pen have become sick within 24 hours.<br>If the criteria are followed, there is adherence to the guideline.	Percentage or proportion ratio in which group treatment has been initiated following the guideline's criteria compared with group treatment not following the guideline's criteria.<br>*What is the appropriate time to measure this? Six or 12 months?*<br>*Is it better to choose specific products, for example oral products compared with parenteral products?*	Value: this outcome indicator measures the criteria for group treatment inaccurately.<br>Registration and measurability: indicator is not measurable because there is not sufficient reliable registration in practice. The number of sick animals when switched to group treatment is not (always) registered precisely by the farmer.<br>Others:<br>The definition of a group treatment was discussed. It was mentioned that it is a group treatment when oral antimicrobials are given.<br>The measurement period needs to be long enough to have a realistic outcome. A period of 18 months is mentioned.<br>Indicator is considered inappropriate.
**10. Antimicrobial use**	The DDDA for weaned piglets also includes antibiotics that are not used for *S. suis*. However, it is one of the few concrete reliable measurements that immediately indicates something about antibiotic use on the farm.<br>Indicator is further developed, see results section of article.	Number indicating the deviation from the DDDA action value for weaned piglets in the Netherlands on selected *S. suis* problem farms.<br>*Is there a seasonal influence in this indicator?*<br>*Can other infections have a big impact?*	Value: outcome indicator that measures adherence to the end objective of guideline, the total antimicrobial use in weaned piglets and how far it differs from the action value.<br>Registration and measurability: this indicator is measurable, but there is no specific registration for *S. suis* separately.<br>Others:<br>The number of animals used for the DDDA measurement is questioned (which period, unreliability of system).<br>If the farm has many disease problems, this indicator could measure antimicrobial use for other diseases although it is noted as *S. suis* in this indicator. This could be solved by choosing only *S. suis* problem farms and therefore assuming that the (most) antimicrobial use is for the treatment *of S. suis.* The measurement period for this indicator is important to minimize seasonal influence on this indicator.<br>Indicator is considered appropriate.

**11. Corticosteroids**	In note 19 of the guideline, it is explained why the use of corticosteroids is advised in individual treatments of *S. suis* piglets. If pig farmers lack knowledge in this regard, corticosteroids may not be used or may be underutilized.<br><br>Indicator is further developed, see results section of article.	The relative proportion of corticosteroids use on the selected *S. suis* problem farms.<br><br>*Should we also look at the quantities of corticosteroids alongside the quantities of antibiotics? If so, how?*<br><br>*Or can we link the quantity of corticosteroids to the individual treatment of piglets? Is this achievable by examining the quantities of 1st choice parenteral antibiotics used for* S. suis *treatments?*<br><br>*Are the antibiotics also used for other conditions? Is it impossible to determine how much of that antibiotic was used by the farmer for* S. suis *treatment?*	Value: outcome indicator that measures whether corticosteroids are used for weaned piglets. It does not measure the volume of the corticosteroids used, but it gives an indication of whether corticosteroids are used at all.<br><br>Registration and measurability: this indicator is measurable but not registered for *S. suis* specifically. However, corticosteroids in weaned pigs are almost exclusively used for the acute treatment of *S. suis*.<br><br>Others:<br><br>Corticosteroids can also be on the list for sows rather than weaned piglets and therefore be missed in this indicator<br><br>Indicator is considered appropriate.
**12. Evaluation of therapy**	The guideline states that all treatments need to be evaluated. On the basis of success or failure, the treatment is finished or a new problem definition is established. Good follow-up is necessary for documenting a farm dossier.<br><br>If the outcome of a treatment is not evaluated, the guideline is not followed.	Percentage of farmers where the treatment has been evaluated and recorded.<br><br>*We can expect the outcome of this indicator to be generally high on average. There may be some exceptions where, due to exceptional circumstances, the therapy has not been evaluated. For example, the farm may have ceased operations. To what extent should this be taken into account in the indicator? Would it therefore be better to assess the indicator on a per-farm basis?*	Value: outcome indicator that measures the administration of an evaluation. In practice, this is not always documented completely but also done verbally and it does not occur after every treatment.<br><br>Registration and measurability: this indicator is measurable if the veterinarian's administration is complete.<br><br>Others:<br><br>In practice, this indicator could measure an administrative action but not the reasoning and effect that is intended. For example, the veterinarian's beliefs about consequences are not measured in this indicator although this is one of the most important behavioral determinants.<br><br>Indicator is considered inappropriate

**13. Death piglets**	If a livestock farmer chooses not to treat the piglets, thus avoiding antibiotic use and/or contacting the veterinarian, the farm can still receive a good score based on the other indicators, despite the high number of dead piglets.<br><br>Mortality of weaned piglets on a pig farm is directly related to its success.	Percentage of mortality of weaned piglets in the past three months.<br><br>*How can veterinarians find out about this? And if the farmer does not contact a veterinarian, to what extent does this indicator reflect veterinarians' adherence to the guidelines?*<br><br>*The question also arises as to whether there are pig farmers who do not treat piglets. Is this common in practice? Is it useful to create an indicator for when a piglet should be euthanized? Farmers are allowed to do this themselves. Does the farmer record this when euthanizing a piglet with* S. suis? *Can we obtain this information?*	Value: outcome indicator that measures dead weaned piglets in a period; this relates to animal welfare. It does not indicate death from *S. suis* only, but also other diseases and cannot directly be linked to adherence to the guideline.<br><br>Registration and measurability: this indicator is measurable but not only for *S. suis* casus and through the farmer (weaned piglets less sold piglets). Therefore, the registration system does not contain enough information for the guideline.<br><br>Others:<br><br>This indicator has a value when farmers have a low antimicrobial use but a high mortality because of *S. suis*. This is seen as unwanted for animal welfare.<br><br>Indicator is considered inappropriate
