# Supplementary material for: The development and application of performance indicators to assess veterinarians’ adherence to the clinical practice Streptococcus suis in weaned pigs guideline
Source: BMC Vet Res. 2025 Feb 25;21:101. doi: 10.1186/s12917-025-04550-0 (PMC11854134; doi:10.1186/s12917-025-04550-0)
Supplement: Supplementary file 2 — Supplementary Material 2 [file 12917_2025_4550_MOESM2_ESM.pdf]

Supplementary Table 2 Summary general discussion points expert panel

General discussion points performance indicators	Explanation
**Data registration**	There is almost no registered data for specific diseases available, and, for a lot of data, veterinarians are dependent on the farmers, who are not always motivated to share their data. Looking up data available from others is time intensive. The amount of time it takes also depends on the veterinarian (how organized and familiar with the management systems or databases is (s)he?).<br>Veterinarians will not (always) be motivated to share this kind of data.
**Benchmarking**	Some indicators can be used for benchmarking or by (official) inspectors, but this is not (always) wanted by the veterinarians in practice.
**Quality guideline**	Maybe the quality of the guideline is not sufficient. The guideline can be evaluated in terms of the outcomes from the indicators developed in this study.
**Other external influencers**	A veterinarian is not the final decision maker at the farm; it is the farmer who decides. Many layers need to be influenced and they are not always under the veterinarian's control. The farmer's influence on many of the developed outcome indicators can be greater than that of the veterinarian.
