# Supplementary material for: The development and application of performance indicators to assess veterinarians’ adherence to the clinical practice Streptococcus suis in weaned pigs guideline
Source: BMC Vet Res. 2025 Feb 25;21:101. doi: 10.1186/s12917-025-04550-0 (PMC11854134; doi:10.1186/s12917-025-04550-0)
Supplement: Supplementary file 3 — Supplementary Material 3 [file 12917_2025_4550_MOESM3_ESM.pdf]

Supplementary Table 3 Questionnaire

**NUMBER LINKED TO PERFORMANCE INDICATOR**	**QUESTION**
	After each farm visit I make a full report
	I try to ensure that my farmers use 1st choice antimicrobials as much as possible
	I do my best to ensure that farmers use individual treatments rather than group treatments as much as possible
	I advise my farmers on euthanasia methods for their animals
	I try to prevent as many problems as possible by identifying risk factors
	I advise my farmers to address risk factors
	I use guidelines as a tool in my daily work
	I deviate from guidelines if I can argument this
	I use the most up-to-date knowledge to advise my farmers
	I know all regulations regarding the use of antimicrobials
Q 3.1	I record the findings from my clinical inspection regarding *S. suis*
Q 3.2	I record an estimate of the number of affected animals regarding *S. suis*
Q 3.3	I record about *S. suis* the (probable) diagnosis
Q 3.4	I record possible additional diagnostics regarding *S. suis*
Q 3.5	I record vaccination status regarding *S. suis*
Q 3.6	I record advice and/or treatment plan regarding *S. suis*
Q 3.7	I record my motivation if I deviate from a 1st choice antimicrobial regarding *S. suis*
Q 3.8	I record the therapy evaluation regarding *S. suis*
Q 3.9	I record the number of animals that will be treated regarding *S. suis*
Q 3.10	I record in which pens and department(s) the animals to be treated are located regarding *S. suis*
Q 5	I advise the farmer to use corticosteroids for piglets with meningitis caused by *S. suis*
	I recommend the farmer to remove sick piglets with meningitis caused by *S. suis* from the group
	I recommend the farmer to give water to sick piglets with meningitis caused by *S. suis* immediately
Q 2	I recommend to the farmer to treat in principle piglets with meningitis caused by *S. suis* with 1st choice antimicrobial
Q 1	I recommend to the farmer to start group treatment if 5% or more piglets with meningitis caused by *S. suis* are affected within five days or 4% or more piglets with meningitis caused by *S. suis* are affected within 24 hours

	I recommend to the farmer to euthanize piglets with severe brain symptoms due to meningitis caused by *S. suis* and piglets that deteriorate within 8 hours
	I recommend to the farmer to euthanize piglets with severe brain symptoms due to meningitis caused by *S. suis* in which no or insufficient recovery occurs within 48 hours
	I recommend to the farmer in piglets with meningitis caused by *S. suis* to do preventive measures to avoid *S. suis* problems
	I recommend to the farmer in piglets with meningitis caused by *S. suis* concrete measures to prevent *S. suis* problems
Q 4.1	I recommend to the farmer in piglets with meningitis caused by *S. suis* if it is not an *S. suis* problem farm, to do a pathological examination twice a year of at least two piglets
Q 4.2	I recommend to the farmer in piglets with meningitis caused by *S. suis* if the farm is an *S. suis* problem farm, to do a pathological examination structurally four times a year of at least two piglets
Q 4.3	I recommend to the farmer in piglets with meningitis caused by *S. suis* if I recommend pathological examination, this includes bacteriological examination & susceptibility determination
	I use the *S. suis* guideline for weaned piglets with meningitis
	I use the *S. suis* guideline for weaned piglets with arthritis
	I use the *S. suis* guideline as a tool in advising my farmers
	I use the checklist in the *S. suis* guideline (or another checklist) to check preventive *S. suis* measures
	I use the *S. suis* guideline because inspectors check me on this
	I use the *S. suis* guideline because it is a good manual
	I use the *S. suis* guideline because it gives me guidance
	I know of each individual farm of my farmers the main risk factors for the *S. suis* problems
	I know of each individual farm of my farmers the antimicrobial use for *S. suis* in weaned piglets
	I identify a farm as an *S. suis* problem farm if the antimicrobial use in weaned piglets is above 20 DDDA due to clinical problems with *S. suis*
	I identify a farm as *S. suis* problem farm if 2nd choice antimicrobials are used to treat weaned piglets with clinical *S. suis* problems
	I do my best to make complete reports
	I do my best to create reports that comply with laws and regulations
	I do my best to ensure that my farmers give piglets with brain symptoms due to *S. suis* corticosteroids
	I do my best to ensure that my farmers use 1st choice antimicrobials
	I do my best to ensure my farmers start group treatments if there are 5% or more affected piglets within five days or 4% or more affected piglets within 24 hours
	I try to ensure that a farm using autovaccination regularly sends piglets for serotyping of *S. suis*
	I try to ensure that a farm using autovaccination sends piglets for serotyping of *S. suis* on a structural basis

I try to ensure that piglets are euthanized in time to avoid unbearable suffering

I try to ensure that I have mapped *S. suis* risk factors of all my farms

I try to ensure that I am up to date with the most current knowledge of *S. suis*

I try to ensure that every farmer removes sick piglets from the flock

I try to ensure that every farmer give waters to sick piglets immediately

My farmers with *S. suis* problems, remove sick piglets directly from the flock

My farmers with *S. suis* problems, give sick piglets due to *S. suis* direct water separately from the group

What percentage (estimated) of your farmers use in the treatment of *S. suis*: corticosteroids

What percentage (estimated) of your farmers use in the treatment of *S. suis*: 1st choice antimicrobials

What percentage (estimated) of your farmers use in the treatment of *S. suis*: 2nd choice antimicrobials

What percentage (estimated) of your farmers used in the treatment of *S. suis*: Individual therapies

What percentage (estimated) of your farmers used in the treatment of *S. suis*: Group treatments

Do you prefer digital or physical meetings for peer consultation?
