# Supplementary material for: The development and application of performance indicators to assess veterinarians’ adherence to the clinical practice Streptococcus suis in weaned pigs guideline
Source: BMC Vet Res. 2025 Feb 25;21:101. doi: 10.1186/s12917-025-04550-0 (PMC11854134; doi:10.1186/s12917-025-04550-0)
Supplement: Supplementary file 4 — Supplementary Material 4 [file 12917_2025_4550_MOESM4_ESM.pdf]

Supplementary Table 4 Number farms per veterinarian

VETERINARIAN	NUMBER *S. SUIS* PROBLEM FARMS IN STUDY
32	5
33	1
35	3
37	4
39	4
41	5
42	2
44	4
45	5
46	5
48	5
49	3
53	4
54	3
55	5
56	4
57	5
58	5
61	2
62	2
63	4
65	1
70	2
72	5
73	5
74	4
75	5
76	5
77	5
79	5
80	1
82	5
83	2
Total	125

The number of farms ranged from a minimum of one to a maximum of five *S. suis* problem farms.
