# Supplementary material for: The development and application of performance indicators to assess veterinarians’ adherence to the clinical practice Streptococcus suis in weaned pigs guideline
Source: BMC Vet Res. 2025 Feb 25;21:101. doi: 10.1186/s12917-025-04550-0 (PMC11854134; doi:10.1186/s12917-025-04550-0)
Supplement: Supplementary file 5 — Supplementary Material 5 [file 12917_2025_4550_MOESM5_ESM.pdf]

Supplementary Table 5 Calculations

Examples of calculations of the five performance indicators are showed in Tables S.5.1–S.5.5

Table S.5.1 Example calculation performance indicator antimicrobial use

EXAMPLE<br>VETERINARIAN X	DATA AT OCT. 31<br>from last 12 months	OUTCOME<br>AND CALCULATION
**FARM 1:**<br>***S. SUIS* PROBLEM FARM**	Total DDDA weaned pigs: 39	
**FARM 2**<br>***S. SUIS* PROBLEM FARM**	Total DDDA weaned pigs: 22	
**FARM 3**<br>***S. SUIS* PROBLEM FARM**	Total DDDA weaned pigs: 12	
**FARM 4**<br>***S. SUIS* PROBLEM FARM**	Total DDDA weaned pigs: 43	
**FARM 5**<br>***S. SUIS* PROBLEM FARM**	Total DDDA weaned pigs: 32	
**TOTAL**	Current action value weaned pigs: 20	((39+22+12+43+32)/5)/20 = 1.48

Calculation in math: ((total DDDA farm 1 + total DDDA farm 2 + total DDDA farm 3 + total DDD farm 4 + total DDD farm 5)/5)/20.

Table S.5.2 Example calculation performance indicator 1st choice antimicrobials

EXAMPLE<br>VETERINARIAN X	DATA AT OCT. 31<br>from last 12 months	OUTCOME<br>AND CALCULATION
**FARM 1:**<br>***S. SUIS*** **PROBLEM FARM**	Total DDDA weaned pigs: 39<br>Percentage 1st choice antimicrobials: 73%	39 x 73 = 2847
**FARM 2**<br>***S. SUIS*** **PROBLEM FARM**	Total DDDA weaned pigs: 22<br>Percentage 1st choice antimicrobials: 50 %	22 x 50 = 1100
**FARM 3**<br>***S. SUIS*** **PROBLEM FARM**	Total DDDA weaned pigs: 12<br>Percentage 1st choice antimicrobials: 10 %	12 x 10 = 120
**FARM 4**<br>***S. SUIS*** **PROBLEM FARM**	Total DDDA weaned pigs: 43<br>Percentage 1st choice antimicrobials: 46 %	43 x 46 = 1978
**FARM 5**<br>***S. SUIS*** **PROBLEM FARM**	Total DDDA weaned pigs: 32<br>Percentage 1st choice antimicrobials: 100%	32 x 100 = 3200
**TOTAL**	(2847 + 1100 + 120 + 1978 + 3200)/(39+22+12+43+32) = 9245/148 = 62.46/100 = 0.62	

*Calculation in math: ((total DDDA farm 1 * %* 1st *choice farm 1) + (total DDDA farm 2 * %* 1st *choice farm 2) + (total DDDA farm 3 * % 1st choice farm 3) + (total DDDA farm 4 * %* 1st *choice farm 4) + (total DDDA farm 5 * %* 1st *choice farm 5))/(total DDDA farm 1 + total DDDA farm 2 + total DDDA farm 3 + total DDDA farm 4 + total DDDA farm 5)*

Table S.5.3 Example calculation performance indicator foundation for the use of 2nd choice antimicrobials

EXAMPLE VETERINARIAN X	DATA AT OCT. 31 from last 12 months	OUTCOME AND CALCULATION
**FARM 1: *S. SUIS* PROBLEM FARM**	Used 2nd choice antimicrobials more than four times.	Denominator = 4
	Last four times they all had a foundation (bacteriological culturing and susceptibility testing or in report)	Numerator = 4
**FARM 2 *S. SUIS* PROBLEM FARM**	Used 2nd choice antimicrobials one time.	Denominator = 1
	Last time there was a foundation (bacteriological culturing and susceptibility testing or in report)	Numerator = 1
**FARM 3 *S. SUIS* PROBLEM FARM**	Used 2nd choice antimicrobials four times.	Denominator = 4
	Last four times, one had a foundation (bacteriological culturing and susceptibility testing or in report)	Numerator = 1
**FARM 4 *S. SUIS* PROBLEM FARM**	Used 2nd choice antimicrobials three times.	Denominator = 3
	All three had a documented foundation (bacteriological culturing and susceptibility testing or in report)	Numerator = 3
**FARM 5 *S. SUIS* PROBLEM FARM**	Used 2nd choice antimicrobials more than four times.	Denominator = 4
	Last four times, one had a foundation (bacteriological culturing and susceptibility testing or in report)	Numerator = 1
**TOTAL**		(4+1+1+3+1)/(4+1+4+3+4) = 10/16 = 0.625

Calculation in math: (foundation farm 1 + foundation farm 2 + foundation farm 3 + foundation farm 4 + foundation farm 5)/(2nd choice farm 1 + 2nd choice farm 2 + 2nd choice farm 3 + 2nd choice farm 4 + 2nd choice farm 5)

Table S.5.4 Example calculation performance indicator bacteriological examination

**EXAMPLE VETERINARIAN X**	**DATA AT OCT. 31 from last 12 months**	**OUTCOME AND CALCULATION**
**FARM 1: *S. SUIS* PROBLEM FARM**	Group treatments: Nov. 4, Jan. 5, Jan. 25, Mar. 4, Aug. 7, and Sept. 9. In quarter: 4 – one, 1 – three; 3 – two; .	Denominator = 4 [a]
	S. suis representatives examined with bacteriological culturing and susceptibility testing: Jan. 5, four piglets. Sept. 9, two piglets.	Numerator = 2
		2/4 = 0.5
**FARM 2 *S. SUIS* PROBLEM FARM**	Group treatments: Dec. 8, Dec. 21, July 4, Sept. 11 and Oct. 2. In quarter: 3 – two; 4 – three.	Denominator = 4 [a]
	S. suis representatives examined with bacteriological culturing and susceptibility testing: Dec. 21, one piglet. Sept. 11, two piglets.	Numerator = 1.5
		1.5/4 = 0.375
**FARM 3 *S. SUIS* PROBLEM FARM**	Group treatments: 0 In quarter: 4 – one.	Denominator = 4 [a]
	S. suis representatives examined with bacteriological culturing and susceptibility testing: 0	Numerator = 0
		0/4 = 0
**FARM 4 *S. SUIS* PROBLEM FARM**	Group treatments: Feb. 2, Mar. 6, Apr. 5, July 5, Sept. 8, Oct. 6, Oct. 16. In quarter: 1 – two; 2 – one; 3 – two; 4 – two.	Denominator = 4
	S. suis representatives examined with bacteriological culturing and susceptibility testing: Oct. 6, two piglets.	Numerator = 1
		1/4 = 0.25
**FARM 5 *S. SUIS* PROBLEM FARM**	Group treatments: Nov. 2, Jan. 15, Jan. 25, Mar. 7, Aug. 9, Sept. 10. In quarter: 1 – three; 3 – two; 4 – one.	Denominator = 4 [a]
	S. suis representatives examined with bacteriological culturing and susceptibility testing: Jan. 15, eight piglets.	Numerator = 1
		1/4 = 0.25
**TOTAL**		(0.5 + 0.375 + 0 + 0.25 + 0.25)/5 = 0.275

Calculation in math: ((bacteriology farm 1/group treatments farm1) + (bacteriology farm 2/group treatments farm 2) + (bacteriology farm 3/group treatments farm 3) + (bacteriology farm 4/group treatments farm 4) + (bacteriology farm 5/group treatments farm 5))/5

[a] If the farm was a problem farm in the past 12 months, the denominator is four, regardless of the number of times an oral treatment was used.

Table S.5.5 Example calculation performance indicator corticosteroids

EXAMPLE<br>VETERINARIAN X	DATA AT OCT. 31<br>from last 12 months	OUTCOME<br>AND CALCULATION
**FARM 1:**<br>***S. SUIS* PROBLEM FARM**	Group treatments:<br>Nov. 4, Jan. 5, Jan. 25, Mar. 4, Aug. 7, Sept. 9.<br>In quarter: 1 – three; 3 – two; 4 – one.	Denominator = 3
	Corticosteroids issued on Jan. 5.<br>In quarter: 1 – one.	Numerator = 1
		1/3 = 0.33
**FARM 2**<br>***S. SUIS* PROBLEM FARM**	Group treatments:<br>Dec. 8, Dec. 21, available 4, Sept. 11, Oct. 2.<br>In quarter: 3 – two, 4 – three.	Denominator = 2
	Corticosteroids issued on Dec. 18, July 1, Oct. 2.<br>In quarter: 3 – one, 4 – two.	Numerator = 2
		2/2 = 1
**FARM 3**<br>***S. SUIS* PROBLEM FARM**	Group treatments:<br>Oct. 3.<br>In quarter: 4 – one.	Denominator = 1
	Corticosteroids issued on July 1.<br>In quarter: 3 – one.	Numerator = 1
		1/1 = 1
**FARM 4**<br>***S. SUIS* PROBLEM FARM**	Group treatments:<br>Feb. 2, Mar. 6, Apr. 5, July 5, Sept. 8, Oct. 6, Oct. 16.<br>In quarter: 1 – two, 2 - one, 3 – two, 4 two.	Denominator = 4
	Corticosteroids issued on Jan. 1, July 7.<br>In quarter: 1 – one, 3 – one.	Numerator = 2
		2/4 = 0.5
**FARM 5**<br>***S. SUIS* PROBLEM FARM**	Group treatments:<br>Nov. 2, Jan. 15, Jan. 25, Mar. 7, Aug. 9, Sept. 10.<br>In quarter: 1 – three, 3 – two, 4 – one.	Denominator = 3
	Corticosteroids issued on Nov. 2, Mar. 3, May 28, Sept. 1.<br>In quarter: 1 – three, 2 – one, 3 – two, 4 – one.	Numerator = 3[a]
		3/3 = 1
**TOTAL**		(0.33 + 1 + 1 + 0.5 + 1)/5 = 0.766

Calculation in math: ((corticosteroids farm 1/group treatments farm1) + (corticosteroids farm 2/group treatments farm 2) + (corticosteroids farm 3/group treatments farm 3) + (corticosteroids farm 4/group treatments farm 4) + (corticosteroids farm 5/group treatments farm 5))/5.

[a] *The numerator can never be greater than the denominator.*
