# Supplementary material for: The development and application of performance indicators to assess veterinarians’ adherence to the clinical practice Streptococcus suis in weaned pigs guideline
Source: BMC Vet Res. 2025 Feb 25;21:101. doi: 10.1186/s12917-025-04550-0 (PMC11854134; doi:10.1186/s12917-025-04550-0)
Supplement: Supplementary file 6 — Supplementary Material 6 [file 12917_2025_4550_MOESM6_ESM.pdf]

Supplementary Table 6 Descriptives and correlations five performance indicators and 16 questions

		M	SD	PI 1	Q 1	PI 2	Q 2	PI 3	Q 3.1	3.2	3.3	3.4	3.5	3.6	3.7	3.8	3.9	3.10	PI 4	Q 4.1	4.2	4.3	PI 5	Q 5
PI 1	Antimicrobial use	0.9	0.7	-																				
Q 1	I recommend to the farmer to start group treatment if 5% or more piglets are affected within five days or 4% or more piglets are affected within 24 hours	3.3	1.5	0.0	-																			
PI 2	The ratio of 1st to 2nd or 3rd choice antimicrobials	0.5	0.3	0.2	-0.1	-																		
Q 2	I recommend to the farmer to treat in principle with a 1st choice antimicrobial	3.7	1.2	0.0	0.1	-0.1	-																	
PI 3	The argumentation for 2nd choice antimicrobials	0.6	0.4	-0.1	0.3	-0.1	0.2	-																
Q 3	The findings from my clinical inspection Q3.1	4.7	0.5	0.2	0.2	0.0	0.1	0.1	-															
I record …	an estimate of the number of affected animals Q3.2	3.6	1.2	0.1	0.1	0.0	0.0	0.2	0.5	-														
	the (probable) diagnosis Q3.3	4.5	0.6	0.0	0.1	-0.1	0.2	0.3	0.5	0.5	-													
	possible additional diagnostics Q3.4	3.8	1.0	0.0	0.0	0.0	0.2	0.1	0.1	0.3	0.4	-												
	the vaccination status Q3.5	3.7	1.3	0.2	0.3	-0.1	0.3	0.2	0.4	0.2	0.4	0.4	-											
	the advice and/or treatment plan Q3.6	4.1	1.0	0.2	0.1	-0.1	0.4	0.2	0.3	0.2	0.4	0.5	0.4	-										
	my motivation if I deviate from a 1st choice antimicrobial Q3.7	4.2	1.2	0.0	0.3	-0.1	0.6	0.2	0.2	0.3	0.2	0.3	0.1	0.5	-									
	the therapy evaluation Q3.8	4.0	1.0	-0.2	0.2	-0.2	0.3	0.4	0.3	0.5	0.5	0.1	0.2	0.2	0.3	-								
	the number of animals that will be treated Q3.9	4.0	1.0	0.0	0.3	-0.1	0.0	0.1	0.1	0.1	0.0	0.1	0.0	0.1	0.1	0.4	-							
	in which pens and department(s) the animals to be treated are located Q3.10	4.0	1.0	0.0	0.2	-0.2	0.2	0.1	0.1	0.1	0.0	0.1	0.1	0.2	0.3	0.4	0.7	-						
PI 4	Bacteriological examination of piglets	0.3	0.2	-0.2	0.2	-0.1	0.1	0.1	0.1	0.1	0.2	0.4	0.3	0.1	0.1	0.1	0.1	0.1	-					
Q 4 I recom-mend to the farmer…	if it is not an *Ss* problem farm, to do a pathological examination twice a year of at least two piglets 4.1	2.2	1.3	-0.3	0.0	-0.1	0.1	-0.1	-0.2	-0.1	0.2	0.3	0.2	0.1	0.1	-0.1	0.0	-0.1	0.3	-				
	if the farm is an *Ss* problem farm, to do a pathological examination structurally four times a year of at least two piglets Q4.2	2.6	1.3	-0.1	0.3	-0.2	0.3	0.1	0.0	0.1	0.2	0.5	0.3	0.2	0.4	0.3	0.2	0.4	0.3	0.4	-			
	if I recommend pathological examination, this includes bacteriological examination and susceptibility determination Q4.3	4.8	0.6	0.1	0.1	-0.2	0.3	0.2	0.2	0.1	0.4	0.2	0.3	0.3	0.4	0.3	0.2	0.4	0.0	0.2	0.3	-		
PI 5	Use of corticosteroids	0.4	0.3	0.0	-0.2	-0.1	0.0	-0.3	-0.3	-0.3	-0.3	-0.2	-0.4	0.0	0.2	-0.1	0.1	0.2	-0.1	0.0	-0.1	0.0	-	
Q 5	I advise the farmer to use corticosteroids	4.2	1.0	0.0	0.0	0.0	0.2	0.0	-0.1	0.0	-0.2	0.0	-0.2	0.1	0.4	0.1	0.3	0.3	-0.1	-0.1	0.2	0.0	0.5	-

PI = Performance indicator, Q = Questionnaire, Ss = *Streptococcus suis*, M = Mean, SD = Standard deviation
